# Study on the role of zoledronic acid in treatment of postmenopausal osteoporosis women

**DOI:** 10.12669/pjms.296.3677

**Published:** 2013

**Authors:** Ma Chao, Qin Hua, Zhou Yingfeng, Wan Guang, Shi Shufeng, Dong Yuzhen, Wang Wei, Tan Haifeng

**Affiliations:** 1 **Ma Chao, Department of Orthopedic Surgery, The First Affiliated Hospital of Xinxiang Medical College, Weihui, China.**; 2 **Qin Hua, Department of Orthopedic Surgery, The First Affiliated Hospital of Xinxiang Medical College, Weihui, China.**; 3 **Zhou Yingfeng, Department of Orthopedic Surgery, The First Affiliated Hospital of Xinxiang Medical College, Weihui, China.**; 4 **Wan Guang, Department of Orthopedic Surgery, The First Affiliated Hospital of Xinxiang Medical College, Weihui, China.**; 5 **Shi Shufeng, Department of Orthopedic Surgery, The First Affiliated Hospital of Xinxiang Medical College, Weihui, China.**; 6 **Dong Yuzhen, Department of Orthopedic Surgery, The First Affiliated Hospital of Xinxiang Medical College, Weihui, China.**; 7 **Wang Wei, Department of Community, Second People’s Hospital of Ji’nan, Ji’nan, China.**; 8 **Tan Haifeng, Department of Digestion, Second People’s Hospital of Ji’nan, Ji’nan, China.**

**Keywords:** Bone mineral density, Fracture, Osteoporosis, Zoledronic acid

## Abstract

***Objective:*** We aimed to assess the role of zoledronic acid (ZOL) on the risk of fracture and bone mineral density (BMD) in women with osteoporosis.

***Methods: ***A double-blind and placebo-controlled design was taken in our study. 327 patients who received an intravenous 5-mg infusion zoledronic acid at day 0, at 12 months were enrolled in treatment group, and the remaining 333 patients who received placebo at the same time of the treatment group were included as control group. The incidence of fracture and BMD in the femoral neck and total hip were assessed.

***Results:*** ZOL group had lower incidence of fracture at any clinical fracture, clinical vertebral fracture, non-vertebral fracture and hip fracture compared with placebo group at the time of one year and three years. We found that the BMD were significantly increased at femoral neck and total hip in ZOL group at the time of one year and three years follow-up when compared with placebo group (P<0.05). The adverse events in the ZOL within three days of drug infusion were significantly higher than the control group, but we did not find significant difference in the serious adverse effect between the two groups.

***Conclusions:*** Zoledronic acid (ZOL) could be used as a safe and effective method for female with osteoporosis.

## INTRODUCTION

Osteoporosis is a skeletal disease that is characterized by compromised bone strength predisposing a person to an increased risk of fracture, and is common in elderly postmenopausal women.^1^ According to the World Health Organization (WHO) data, osteoporosis affects approximately 75 million person in Europe, the US, and Japan, and 9 million new fractures were caused by osteoporosis^2^ Osteoporosis-related fractures are associated with significant morbidity, increased mortality and enormous financial costs.^3^

Teriparatide, calcitonin, alendronate and stontim ranelate have proved to be the standard treatment of osteoporosis.^4^^-^^6^ Previous clinical studies indicated that nitrogen-containing bisphosphonates can inhibit bone resorption, keep bone mass and decrease the risk of osteoporosis-related fractures fractures^7^^,^^8^ Oral bisphosphonates have been shown to increase bone mineral density (BMD).^9^^,^^10^ Zoledronic acid (ZOL) is an intravenous, aminobisphosphonate with a high affinity for mineralized bone, which could increase patients’ compliance with bisphosphonate therapy and thus improve the clinical outcome. ZOL 5mg has been reported to decrease the risk of fracture and increase the bone mineral density among postmenopausal osteoporosis in several developed countries.^11^^,^^12^ However, there were few studies on the effectiveness and safety of intravenous ZOL in Chinese postmenopausal osteoporosis women. Therefore, we aimed to assess the role of ZOL on the risk of fracture and BMD in women with osteoporosis.

## METHODS

***Study population:*** A double-blind and placebo-controlled design was taken in our study. A total of 660 female patients who were diagnosed with osteoporosis were included from The First Affiliated Hospital of Xinxiang Medical College and the Second People’s Hospital of Ji’nan between January 2009 and May 2012. Patients with secondary osteoporosis or other diseases which were known to affect bone metabolism were excluded. Patients taking anabolic steroids, sodium fluoride, and parathyroid or growth hormone within 6 months were also excluded. Patients who had malignant neoplasm, serum calcium more than 11.0 mg/dl, or untreated hypocalcemia were also excluded. All patients signed the informed consent.

***Techniques:*** 660 female patients were randomly divided into two groups. 327 patients who received an intravenous 5-mg infusion zoledronic acid at day 0, at 12 months were included in treatment group, and the remaining 333 patients who received placebo (Activated Vitamin D3, 0.25 mg) at the same time of the treatment group were included as control group. All patients were supplemented with 600-1500 mg elemental calcium and 400-1200 IU vitamin D every day. Patients were followed up for two years with telephone interviews and clinic visits at 12 and 36 months.

***Fracture and BMD measurement:*** All the fractures were assessed by the Genet semi-quantitative method.^13^ Clinical fracture reports were obtained from the routine examination by radiologic or surgical procedure report or a copy of the radiograph. BMD in the femoral neck and total hip was measured by Hologic Dual Energy X-ray Absorptiometry (Hologic, Waltham, MA, USA) at 12 and 36 months.

***Safety assessment:*** All adverse events and serious adverse events were recorded by physical examination and regular measurement of vital signs, hematologic, blood chemical and urinary values. Adverse events were assessed and categorized by the Medical Dictionary for Regulatory Activities.^14^ The most common adverse events were reported within three days of infusion 5-mg infusion zoledronic acid.

***Statistical analysis:*** Efficacy and safety parameters were compared between ZOL and control groups by t-test for continuous variables or chi-square test for categorical variables. The incidence of fractures was expressed as percentage. The BMD change was evaluated as the mean percentage change from baseline. All statistical analyses were conducted by SPSS 11.0 software (SPSS, Chicago, IL), *P* value<0.05 was regarded as statistically significant and all tests were two-sides.

## RESULTS

Among 660 patients, 327 patients were randomized in zoledronic acid group and 333 patients in placebo group. The clinical characteristics are shown in Table-I. The average age in ZOL and placebo groups was 54.6±7.3 years and 55.3±7.5 years, respectively. The BMI, femoral neck bone mineral density, vertebral fractures before treatment, and T score at femoral neck did not show significant difference between zoledronic acid and placebo groups.

**Table-I:** Characteristics of included patients

***Items***	***Zoledronic acid***	***%***	***Placebo***	***%***	***Statistical value***	***P value***
**Age (years)**	**54.6±7.3**		**55.3±7.5**		**0.44**	**0.77**
**Body mass index(kg/m** ^2^ **)**	**24.2±0.5**		**24.3±0.5**		**0.11**	**0.06**
**Femoral neck bone mineral density, g/cm** ^2^	**0.56±0.14**		**0.55±0.15**		**0.58**	**0.29**
**Vertebral fractures before treatment**						
**Yes**	**184**	**56.3**	**182**	**54.7**		
**No**	**143**	**43.7**	**151**	**45.3**	**0.08**	**0.77**
**T score at femoral neck**						
**≤-2.5**	**151**	**46.3**	**152**	**45.7**		
**-2.5 to -1.5**	**168**	**51.3**	**172**	**51.6**		
**≥-1.5**	**8**	**2.4**	**9**	**2.7**	**0.11**	**0.95**

**Fig.1:**
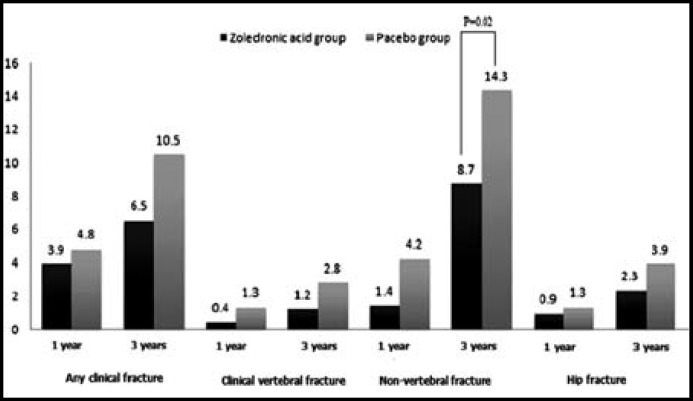
Incidence of fractures at the time of 12 months and 24 months

Fig.1 shows the incidence of fractures at the time of 12 months and 36 months. We found ZOL group had lower incidence of fracture at any clinical fracture, clinical vertebral fracture, non-vertebral fracture and hip fracture compared with placebo group. However, only the incidence of non-vertebral fracture in the ZOL group was significantly lower than the ZOL group (14.3% vs 8.7%, *P*<0.05), and a reduction of 46% in the risk of fracture when compared with placebo group (OR=0.55, 95% CI=0.33-0.93).

Table-II shows the percentage change in Bone Mineral Density between the two groups. We found that the BMD was significantly increased at femoral neck and total hip in ZOL group at the time of one year and three years follow-up when compared with placebo group (*P*<0.05). The mean difference of femoral neck BMD percentage change of ZOL versus placebo was 1.81(1.54-2.26) at one year follow-up, and was 3.65(3.31-4.04) at 3 years follow-up. Meanwhile, the mean difference of total hip BMD was 2.12(1.78-2.45) at one year follow-up, and was 4.26(3.80-4.81) at three years follow-up.

**Table-II:** Percentage change in Bone Mineral Density in the two groups

***Bone Mineral Density***	***Relative treatment differences (95% CI)*** ^1^	***P value***
**Femoral neck BMD**		
**1 year**	**1.81(1.54-2.26)**	**<0.05**
**3 years**	**3.65(3.31-4.04)**	**<0.05**
**Total hip BMD**		
**1 year**	**2.12(1.78-2.45)**	**<0.05**
**3 years**	**4.26(3.80-4.81)**	**<0.05**

1. The mean percentage difference of Bone Mineral Density of zoledronic acid group versus placebo

The incidence of adverse evidence of in the ZOL group was 84.2%, and 81.6% in the placebo group. There was no significant difference in the adverse events between the two groups. The adverse events in the ZOL within three days of drug infusion were significantly higher than the control group (42.3% versus 20.7%). The common adverse events in the two groups were back pain, urinary tract infection, hypertension, nasopharyngitis, arthralgia, pyrexia, myalgia, headache and influenza-like symptoms. Three patients in the ZOL treatment group and one patient in the control group showed serious cardiac symptoms, and no significant difference was found between them.

## DISCUSSION

In the present study, intravenous 5-mg infusion zoledronic acid for 12 months significantly increased the BMD at femoral neck and total hip, and decreased the risk of any clinical, clinical vertebral and non-vertebral as well as hip fracture in postmenopausal women.

Our study found that intravenous 5-mg infusion zoledronic acid at the baseline and at 12 months could significantly the risk of non-vertebral fracture among females who were diagnosed with osteoporosis. Previous several studies indicated that oral risedronate treatment could reduce the risk of vertebral and nonvertebral fractures.^15^^,^^16^ Reginster indicated that the oral bisphosphonate could reduce the risk of new vertebral fracture by 49% over 3 years when compared with placebo control, and the risk of non-vertebral fractures was reduced by 33% compared with placebo control.^15^ Harris reported that oral risedronate could decrease the risk of new vertebral fractures and non-vertebral fractures by 41% and 39%, respectively.^16^ Our results are in line with the previous studies.

In our study, we found that the BMD was significantly increased at femoral neck and total hip in ZOL group over three years when compared with placebo group. Previous experimental study indicated that zoledronic acid administration could increase bone mineral density in the trabecular bone compared with control group.^9^ A previous clinical HORIZON trail with 107 patients indicated that intravenous zoledronate therapy significantly increased BMD of lumbar spine over 3 years.^10^ Another clinical trial conducted in China reported that zoledronic acid administration once a year could increase BMD and reduce the serum bone turnover metabolism.^17^ Boonen et al reported a single 15-minute infusion of zoledronic acid (5 mg) is associated with a significant improvement in BMD, and was not association with serious adverse events when compared with placebo-controlled trial.^18^ However, another study with 7765 postmenopausal osteoporosis women indicated that intravenous 5-mg infusion zoledronic acid did not increased the femoral neck BMD compared with control.^19^ The possible discrepancy of the results may be induced by different backgrounds of cases, sample size, sample size and etc. Therefore, the effect of ZOL on women with osteoporosis should be confirmed in further large sample size studies.

Our study showed that the adverse events in the ZOL within three days of drug infusion were significantly higher than the control group, and the main reason of the higher risk of adverse effect in the ZOL group was the first-dose acute-phase reaction. The most common adverse events of the first-dose acute-phase reaction were back pain, urinary tract infection, hypertension, nasopharyngitis, arthralgia, pyrexia, myalgia, headache and influenza-like symptoms. However, we did not find significant difference in the serious adverse effect between the two groups, which indicates ZOL is a safe and efficacy treatment for women with osteoporosis.

In conclusion, the present three -year follow-up study indicated that intravenous 5-mg infusion zoledronic acid at the baseline and at 12 months could significantly the risk of non-vertebral fracture among females who were diagnosed with osteoporosis, and increase the BMD of femoral neck and total hip over three years when compared with placebo group. In additional, this treatment did not have serious drug-related adverse effects, and intravenous 5-mg infusion ZOL could be used as a safe and effective method for female with osteoporosis.
